# Successful Case of Ligature of the Internal Iliac Artery, for the Cure of Gluteal Aneurism

**Published:** 1828-07

**Authors:** S. Pomeroy White

**Affiliations:** Surgeon, Hudson, N. Y.


### GLUTEAL ANEURISM.

*Successful Case of Ligature of the Internal Iliac Artery, for the cure of Gluteal Aneurism.* By S. Pomeroy White, Surgeon, Hudson, N. Y.

In the early part of October last, Jacob Van Volkenburg, aged sixty, by trade a tailor, came to Hudson for the purpose of obtaining surgical advice. He presented to our view a tumor about the size of a child's head, located upon his left hip, directly over the sciatic notch. He stated that it was of ten months standing, and that he experienced no pain from it. His general health had been good, except that he suffered from rheumatism. Upon making an examination of the tumor, the skin was found not discoloured; fluctuation was perceptible, but there was no pulsation. The absence of the last symptom rendered it difficult to decide upon the nature of the case, and accordingly we postponed giving an opinion until a consultation could be held with Dr. Hicks. The Doctor stated that the tumor could be removed by pressure, when small, but, from other cases he had seen of a similar kind, he presumed that it contained pus. He proposed opening it, and, as that was the only way of ascertaining unequivocally the nature of the disease, we acquiesced. He accordingly punctured it, and nothing but florid blood made its appearance. A probe was passed in, and an aneurismal sac was found, about five inches deep. It was also discovered that the parietes of the sac were very firm and unyielding, which accounted for the absence of pulsation. After allowing a pint of blood to be discharged, the orifice was closed with a suture and adhesive plaster. It was observed that after this, and also subsequent discharges of blood, that the sac would fill again, and the tumor resume its usual dimensions. The disease was supposed to be produced by repeated falls upon his left nates. He was a man of rather intemperate habits, and, when stupified by liquor, would fall, as they expressed it, like a log. Being also lame in his left limb from rheumatism, he consequently was most liable to fall upon his left nates.

The consultation board having unanimously concluded that the disease was aneurismal, it then became a question whether it was most for the interest of the patient to tie the gluteal or internal iliac artery. As a precedent for the former, we had Mr. John Bell's famous case. In that, with all his dexterity, he barely succeeded in saving his patient, in consequence of the hemorrhage, though some allowance must be made for his usual hyperbole. My father, also, stated in the consultation, that he had been under the necessity of tying the gluteal at the notch, but under the existing circumstances of this case he would not recommend that operation. Indeed, the inability of compressing the artery above the disease, the possibility of the patient dying of hemorrhage before the artery could be secured, and the probability of the artery being diseased within the notch, were insuperable objections to tying the gluteal. On the other hand, we had an encouraging, although a solitary* precedent, in the case of Mr. Stevens, of the Island of Santa Cruz. There the disease was the same, and the application of a ligature to the internal iliac proved successful. The operation of tying the internal iliac was accordingly concluded upon, though the patient's age was considered as a circumstance that would operate against its success. He at first declined having the operation performed, but, as profuse hemorrhage repeatedly supervened, he became weak and alarmed, and requested us to pursue whatever course we deemed most expedient.

On the 23d of October, he was laid upon the table, and an incision was made of a semicircular form, commencing two inches to the left of the umbilicus, and ending near the external ring. It was seven inches in length, and the convexity of it was towards the ileum.

After dividing the skin, cellular substance, and superficial fascia, it became necessary to secure a few small arteries. The tendon of the external oblique being exposed, was next divided, and then the internal oblique and transversalis with its fascia. The peritoneum, which now presented, was detached from the iliacus internus and psoas magnus muscles with the fingers, and was pressed with its contents towards the right hypochondriac region, by the assistance of my father. The external iliac was immediately felt, and, by passing the finger towards the sacro-iliac symphysis, the internal iliac was distinctly recognised. The artery was then exposed with the handle of the scalpel, and the ligature passed under with the Philadelphia needle, one inch from the bifurcation. Instead, however, of drawing up the needle part with the hook, I found it more convenient to take it with the dressing forceps. One ligature being passed, it was found necessary, from the great depth of the parts, (being about five inches,) to pass down the knot with Dr. A. E. Hosack's knot applicator. The ligature was then firmly tied, and the parts were brought together with sutures and adhesive plaster.

In this operation the same difficulty existed as in the case of

* I have, since the operation, understood that the internal iliac has been tied by a Dr. Atkinson, in England, but without success.

ligature of the common iliac, by my distinguished friend Dr. Mott, of New York,—viz. the constant protrusion of peritoneum, from abdominal compression created by the struggles of the patient.

Some pain in the bowels and fever came on a few days after the operation, which was removed by venesection and a laxative. Union by the first intention had taken place to a considerable extent at the first dressing on the eighth day. A considerable quantity of pus was discharged during the first four weeks, at the expiration of which time the ligature came away. The tumor has discharged its contents gradually, and the parts have assumed their natural appearance. The patient has so far recovered his usual state of health, as to be able to walk about his neighbourhood.*

